# Serum Catestatin Levels and Arterial Stiffness Parameters Are Increased in Patients with Inflammatory Bowel Disease

**DOI:** 10.3390/jcm9030628

**Published:** 2020-02-26

**Authors:** Piero Marin Zivkovic, Andrija Matetic, Ivana Tadin Hadjina, Doris Rusic, Marino Vilovic, Daniela Supe-Domic, Josip Andelo Borovac, Ivana Mudnic, Ante Tonkic, Josko Bozic

**Affiliations:** 1 Department of Gastroenterology, University Hospital of Split, 21000 Split, Croatia; piero.zivkovic@gmail.com (P.M.Z.); ihtadina@gmail.com (I.T.H.); jobo5k@yahoo.com (A.T.); 2 Department of Pathophysiology, University of Split School of Medicine, 21000 Split, Croatia; andrija.matetic@gmail.com (A.M.); marino.vilovic@mefst.hr (M.V.); josip.borovac@me.com (J.A.B.); 3 Department of Cardiology, University Hospital of Split, 21000 Split, Croatia; 4 Department of Pharmacy, University of Split School of Medicine, 21000 Split, Croatia; doris.rusic@mefst.hr; 5 Department of Medical Laboratory Diagnostics, University Hospital of Split, 21000 Split, Croatia; daniela.supedomic@gmail.com; 6 Department of Health Studies, University of Split, 21000 Split, Croatia; 7 Department of Pharmacology, University of Split School of Medicine, 21000 Split, Croatia; ivana.mudnic@mefst.hr; 8 Department of Internal Medicine, University of Split School of Medicine, 21000 Split, Croatia

**Keywords:** catestatin, inflammatory bowel disease, arterial stiffness

## Abstract

Catestatin (CST) is an important peptide in the pathophysiology of chronic inflammatory disorders. However, clinical studies on inflammatory bowel disease (IBD) patients are lacking. Our goal was to investigate CST concentrations in IBD patients compared to healthy subjects. Additionally, we aimed to determine arterial stiffness parameters in relation to CST. This cross-sectional study compared 80 IBD patients (45 Crohn’s disease (CD) and 35 ulcerative colitis (UC) patients) with 75 control subjects. Serum CST levels were significantly higher in the IBD group compared to control subjects (11.29 ± 9.14 vs. 7.13 ± 6.08 ng/mL, *p =* 0.001) and in the UC group compared to CD patients (13.50 ± 9.58 vs. 9.03 ± 6.92 ng/mL, *p =* 0.021), irrespective of age and BMI. IBD patients exhibited significantly higher values of heart rate adjusted central augmentation index (cAIx-75) (14.88 ± 10.59 vs. 6.87 ± 9.50 %, *p <* 0.001) and pulse wave velocity (PWV) (8.06 ± 3.23 vs. 6.42 ± 1.47 m/s, *p <* 0.001) compared to control group. Furthermore, PWV was the only significant independent correlate of CST (*B =* 1.20, *t =* 4.15, *p <* 0.001), while CST, PWV, cAIx-75, high-sensitivity C-reactive protein and BMI were significant predictors of positive IBD status (1.089 (1.022–1.161), 1.515 (1.166–1.968), 1.060 (1.024–1.097), 1.458 (1.116–1.906), 0.793 (0.683–0.920), respectively). Serum CST levels were significantly higher in IBD patients compared to controls and an independent positive correlation of CST with PWV existed. Therefore, it is possible that CST could have a role in the complex pathophysiology of IBD and its cardiovascular complications.

## 1. Introduction

Inflammatory bowel disease (IBD) is a chronic, relapsing disorder that predominately affects small and large intestine, and its two main manifestations are in the form of ulcerative colitis (UC) and Crohn’s disease (CD) [1]. IBD is believed to be caused by a combination of several predisposing conditions, such as genetic structure, abnormal immunological response and exposure to environmental factors [2]. Furthermore, it is considered that its hallmark characteristic, chronic intestinal inflammation, occurs when an abnormal immunological response causes microvascular endothelial cell damage [3].

Patients with IBD generally have a lower prevalence of classical cardiovascular risk factors, such as obesity, dyslipidemia, and hypertension, but an increased cardiovascular risk in general when compared with healthy population [4,5]. The probable reason for this paradox lies in chronic inflammation effect on arteries, and consequently, the heavily connection of IBD with a number of cardiovascular disorders, including atherosclerosis, endothelial dysfunction and increased arterial stiffness [6,7,8]. In addition, the adverse inflammatory effect on arteries was further confirmed with a recent systematic review by Zanoli et al., that reported a significant increase in pulse wave velocity (PWV), a golden standard for estimation of arterial stiffness, in patients with IBD. Increased arterial stiffness likely presents the initiation and/or progression of atherogenesis and arterial hypertension [9].

Catestatin (CST) is a novel multifunctional peptide proteolytically cleaved from chromogranin A (ChgA), that primarily acts as an inhibitor of catecholamine secretion in vitro in cultured cells and in vivo from mouse adrenal medulla, and as stimulator of histamine release [10,11,12]. Studies implicate its connection with multiple functions throughout various systems, including vasodilatation [13], immunoregulation [14], insulin resistance [15,16,17], antimicrobial effect [18,19] and obstructive sleep apnea [20]. Furthermore, it has been reported to be a key regulator of cardiovascular function, with cardioprotective effects including exhibiting the trough suppression of atherosclerosis [21], negative inotropic and lusitropic cardiac effects [22], inhibition of coronary vasoconstriction [21], a reduction in oxidative stress in ischemic-reperfused myocardium [22], a decrease in endothelial inflammation and regulation of blood pressure [10,23,24,25]. CST is believed to have an important role in the pathophysiology of hypertension, heart failure, reperfusion injury and coronary heart disease [25,26,27]. Intestinal inflammation has been associated with dysfunctional ChgA production [28]. Consequently, several experimental studies on animal colitis model investigated the relationship of intestinal inflammation and CST secretion [29,30,31,32]. Rabbi et al. evaluated the impact of CST on intestinal microbiota in mice and revealed significant changes that could potentially lead to development of new options in treating IBD patients [29]. Furthermore, a few experimental studies have reported that an injection of human CST significantly attenuates intestinal inflammation and severity of inflammatory reactivation through down-regulation of macrophages, pro-inflammatory cytokines and pathways, including interleukin 6 (IL-6), interleukin 1β, tumor necrosis factor α (TNF-α) and signal transducer and activator of transcription 3 (STAT3) dependent pathway [30,31,32]. Muntjewerff et al. even considered CST as an important potential therapeutic modality for various inflammatory and metabolic diseases [33].

Until this point, and to the best of our knowledge, only one study reported serum CST levels in small subset of IBD patients. However, they did not assess any interaction of CST and other clinically relevant factors and biochemical parameters, except C-reactive protein [31]. In addition, there are no published studies that explored the association of CST levels with arterial stiffness parameters, an important surrogate marker of increased cardiovascular risk, in IBD patients. Therefore, the aim of this study was to determine serum CST levels in IBD patients and to investigate its association with arterial stiffness parameters, as well as other biochemical and clinical parameters.

## 2. Experimental Section

### 2.1. Study Design

The study was organized as a cross-sectional observational study. It was conducted at the Department of Gastroenterology, University Hospital of Split, and the Department of Pathophysiology, University of Split School of Medicine over a period from December 2017 to June 2018. 

### 2.2. Ethical Considerations

The study protocol was approved by the Ethics Committee of the University of Split School of Medicine (Class: 003-08/17-03/0001; Registration number: 2181-198-03-04-17-0061; 27 November 2017) and Ethics Committee of the University Hospital of Split (Class: 500-03/17-01/86; Registration number: 2181-147-01/06/M.S.-17-2; 23 November 2017). Written informed consent was obtained from all the study participants prior to the inclusion in the study. The study was conducted in accordance with the statements of the Nuremberg code and ethical principles for medical research as defined by the Declaration of Helsinki and its amendments. 

### 2.3. Subjects

This study included 80 patients with IBD, 45 of which had CD and 35 UC. Patients were compared with control group which consisted of 75 healthy subjects. All participants were above 18 years of age and underwent the full study protocol, except fecal calprotectin assessment which was measured only in the patient group. The patients with IBD were recruited from the outpatient clinic of the Department of Gastroenterology, University Hospital of Split. At the beginning of the study, 100 patients were screened for inclusion in the study and 86 patients were found to be eligible for inclusion of the study. Among mentioned, two participants were excluded due to a sudden disease worsening, two due to inability to schedule an appointment and two due to refusal of further procedures (Supplementary Figure S1).

IBD diagnosis was made in accordance with the latest guidelines of the European Crohn’s and Colitis Organization and the European Society of Gastrointestinal and Abdominal Radiology [34]. The following inclusion criteria were used: disease duration of at least one year and stable disease activity in the previous 3 months. Exclusion criteria were arterial hypertension; diabetes mellitus and/or use of antidiabetic medication; history of heart failure; history of cardiovascular or cerebrovascular events; known peripheral artery disease; chronic kidney disease; pulmonary disease; liver disease; chronic inflammatory disorders other than IBD; malignant disease and use of local/systemic corticosteroids in the past three months. Control subjects were recruited from the healthy blood donors, acquaintances of the investigators and healthy volunteers from the local primary health care center. All control subjects were screened for the presence of the Rome IV criteria for inflammatory bowel syndrome, as well as any other abdominal symptom suggestive of lactose and gluten intolerance, or any type of gastrointestinal symptoms. If any of these symptoms or conditions were present, we excluded the subjects from the control group.

### 2.4. Clinical Assessment and Anthropometric Measurements

All subjects underwent a detailed physical examination and anthropometric data assessment. A calibrated medical scale with an altitude meter (Seca, Birmingham, UK) was used to measure body mass and height. Body mass index (BMI) was calculated by dividing the value of body mass (kg) by the squared value of height (m^2^). The waist circumference was measured at the level of the midline between the bottom line of the rib arch in the mid-axillary line and the tip of the iliac crests, with the patient standing upright. The hip circumference was measured at the level of the largest circumference of the gluteal muscles, above the line joining the great trochanters of the femur. The waist-hip ratio (WHR) was calculated by dividing waist circumference with the hip circumference value. A standard mercury sphygmomanometer, with a suitable arm, was used to measure arterial blood pressure, and values were obtained after a minimum of two measurements. The subjects were seated, with the upper arm at heart level, and had a 10-min rest period before the measurement. Finally, relevant clinical data were extracted from patients’ medical records. Anamnestic data, including tobacco, coffee or alcohol consumption, were taken from all study participants. 

### 2.5. Biochemical Analysis

Blood samples were collected from all participants in the morning period, after at least 10-hour fast. All samples were analyzed in a single laboratory by an experienced biochemist following the same standard procedure. The biochemist was blinded for the participant’s assignment to experimental or control group. Blood was drawn from a polyethylene catheter inserted into the antecubital vein. Serum CST (Cat. no. EK-053-27CE, EIA kit, Phoenix Pharmaceuticals Inc., Burlingame, CA, USA) levels were determined by an enzyme-linked immunosorbent assay (ELISA). Manufacturer reported sensitivity of the assay kit for CST of 0.05 ng/mL with a linear range of 0.05–0.92 ng/mL, and a cross-reactivity with endogenous human CST peptide of 100% (intra-assay and inter-assay coefficients of variability were <10% and <15%, respectively). Fecal calprotectin concentrations were measured by turbidimetric method (Beckman Coulter AU 680), while plasma high sensitivity C-reactive protein (hs-CRP) levels were determined using a latex turbidimetric method (Abbott Laboratories, Chicago, USA). Other biochemical analyses, including lipid parameter measurements, were measured by standard laboratory methods.

### 2.6. Assessment of Disease Severity

Disease activity in patients with IBD was assessed by two experienced gastroenterology specialists independently, using well-established clinical and endoscopic scoring systems. Where the two differed in scores, a consensus was made. Disease activity among patients with UC was assessed using the UC endoscopic index of severity (UCEIS), Mayo endoscopic score (MES) and Mayo score/disease activity index for UC (Mayo/DAI) [35,36,37]. Disease severity in patients with Crohn’s disease was assessed using simple endoscopic score (SES-CD), the Crohn’s disease activity index (CDAI) and the Harvey Bradshaw index (HBI) [37,38,39]. According to the latest ECCO guidelines, clinical indices should be carefully used since they are not well-validated in the clinical practice and discrepancies could be seen [34]. Therefore, we have stratified our patients using only endoscopic index score, while clinical index scores were only descriptively reported. We have stratified UC and CD group based on the endoscopic disease activity score into different categories. A total of 3 patients have been excluded from the sub-analysis (2 from UC and 1 from CD) due to insufficient number of cases per subgroup. 

### 2.7. Measurements of Arterial Stiffness

Applanation tonometry, a widely used standard non-invasive method with a strong correlation to invasively obtained values, has been used for arterial stiffness assessment [39]. Readings were obtained by SphygmoCor (Version 8.1; AtCor Medical, Inc., Sydney, Australia) [40]. Measurements were taken with participants resting in a supine position, in the morning period after an overnight fast, in a temperature-controlled room (constant temperature between 22 °C and 24 °C). Repeated measurements were taken to obtain a minimum of two satisfactory readings according to the built-in quality control requirements. Quality score ranged from zero to 100. Measurements with quality score of less than 90 were repeated in order to acquire acceptable results. Two trained operators performed all vascular measurements. Intra- and inter-operator variability of the PWV measurement were 0.08 ± 0.05 m/s and 0.17 ± 0.10 m/s, respectively. Readings of the carotid to femoral PWV and pulse wave analysis (PWA) were obtained.

During the measurement of the PWV, a tonometric transducer is placed on the carotid and femoral artery. Distance measurement was taken in a direct line between the points of strongest pulsation of the right carotid/femoral artery and supra-sternal notch, by simple flexible measurement scale device. The intersecting tangent algorithm has been used in the SphygmoCor software and the direct carotid-femoral distance has been corrected for 0.8 to obtain relevant aortic PWV values [40]. The applanation tonometric readings of the carotid and femoral pulse were acquired sequentially in order to do the measurement by a single operator. Device calibration was made based on an average of two consecutive blood pressure measurements. For the assessment of the PWA, a tonometric transducer is placed on the subject’s radial artery. PWA included peripheral and central augmentation index (pAIx, cAIx), central pulse pressure (cPP), central systolic blood pressure (cSBP), central diastolic blood pressure (cDBP), central mean blood pressure (cMBP), peripheral pulse pressure (pPP), peripheral systolic blood pressure (pSBP), peripheral diastolic blood pressure (pDBP) and peripheral mean blood pressure (pMBP). The values of cAIx were standardized to a resting heart rate of 75 beats-per-minute (cAIx-75) by the SphygmoCor software [41].

### 2.8. Statistical Analysis

Statistical software SPSS (IBM Corp, Armonk, NY, USA; version 25) and SigmaPlot (Systat Software Inc., San Jose, CA, USA; version 14) for Windows was used for statistical data analysis and graph design. Normality of data distribution was first assessed graphically (histograms, Q-Q plots, box plots) and subsequently by the Kolmogorov–Smirnov test. Data were considered normally distributed if all graphical and analytical tests were concordant, whereas any inconsistency was analysed as non-parametric data. Data were expressed as means ± standard deviation (SD) or median (interquartile range) for continuous variables and as whole numbers and percentages for categorical variables. The Student’s t-test, Mann–Whitney U test and Chi-square test were used for the analysis of independent continuous data and categorical data between the IBD and control group, respectively. Fisher’s exact test was used for categorical data comparison in the case of small expected value in any cell (<5). Analysis of covariance (ANCOVA) was used to compare group differences in CST and arterial stiffness parameters controlling for important covariates including age and BMI. The aforementioned variables were included in the ANCOVA model due to their relevance to CST levels and arterial stiffness. All the assumptions for ANCOVA analysis were satisfied. Levene’s test for the equality of variances was performed prior to the ANCOVA analysis. Moreover, to assess the relationship between different parametric and non-parametric variables Pearson and Spearman correlation methods have been used, respectively. Multiple linear regression analysis with enter selection algorithm was used to determine the relative importance of independent variables (age, BMI, waist circumference, cAIx-75 and PWV) in prediction of CST levels. Finally, predictors of positive IBD status were determined by binomial logistic regression analysis. An odds ratio with a 95% confidence interval has been used for the presentation of the logistic regression analysis data (OR (95% CI)). The statistical significance was defined as *p <* 0.05.

### 2.9. Sample Size Analysis

Sample size analysis has been conducted a priori to the study onset, using a data from a pilot study on 15 healthy subjects and 15 IBD patients. Firstly, graphical estimation has been using nomogram with subsequent confirmation by specific sample size formula. The analysis was calculated using the data on a CST variable. However, sample size of 67 participants per group has been indicated as clinically significant to detect standardized difference of 0.50, with 80% power using a cutoff for statistical significance of 0.05 (confidence interval 95%).

## 3. Results

### 3.1. Basic Anthropometric and Related Characteristics of the Study Sample

There was no significant difference in anthropometric characteristics between studied groups, except in body weight (75.03 ± 14.10 vs. 80.47 ± 13.28 kg, *p =* 0.015) and body height (176.41 ± 9.27 vs. 180.03 ± 9.18 cm, *p =* 0.017) which were lower in IBD patients. Furthermore, IBD patients had higher prevalence of family history of IBD (20.0%, *n =* 16 vs. 4.0%, *n =* 3, *p =* 0.003), while there was no difference in other anamnestic data and habits (Table 1).

Baseline differences in laboratory parameters between the studied groups are shown in Supplementary Table S1.

### 3.2. Basic Characteristics of the IBD Group

There was no significant difference in most anthropometric and disease-related parameters between UC and CD patients. However, CD group did have higher proportion of extraintestinal manifestations (62.8%, *n =* 27 vs. 18.2%, *n =* 6, *p <* 0.001) and IBD-related surgical procedures (35.6%, *n =* 16 vs. 2.9%, *n =* 1, *p <* 0.001).

Furthermore, there was no difference in pharmacologic therapy modalities between IBD subgroups except in aminosalicylates, which were more used in UC patients (85.7%, *n =* 30 vs. 60.0%, *n =* 27, *p =* 0.012).

According to the international endoscopic scoring systems, UC patients had a moderate to severe endoscopic form of disease (UCEIS; MES), while CD patients mostly had moderate endoscopic disease activity (SES-CD) (Table 2). There was no difference in selected variables according to the different disease activity subgroups of UC and CD stratified by endoscopic scores (Supplementary Table S2 and S3).

There was no significant difference in most laboratory parameters between IBD subgroups except in hs-CRP levels which were higher in CD patients (16.43 ± 12.93 vs. 2.33 ± 2.19 mg/L, *p <* 0.001), while UC patients had higher levels of lipid parameters (Supplementary Table S4).

### 3.3. Serum CST Levels

CST levels were significantly higher in the IBD group in comparison to control subjects (11.29 ± 9.14 vs. 7.13 ± 6.08 ng/mL, *p =* 0.001). The difference remained significant after adjustment for age and BMI in ANCOVA analysis (*p =* 0.001) (Table 3).

Furthermore, UC group had significantly higher levels of CST in comparison to CD patients (13.50 ± 9.58 vs. 9.03 ± 6.92 ng/mL, *p =* 0.021), which remained significant after ANCOVA analysis (Table 3). Moreover, there was no difference in CST levels between IBD subgroups based on median disease duration (Supplementary Table S5). Finally, there was no difference in CST levels between IBD subgroups based on biological therapy (11.21 ± 8.22 ng/mL in biological therapy group vs. 11.42 ± 10.72 ng/mL in non-biological therapy group, *p =* 0.923) (Supplementary Table S6).

### 3.4. Arterial Stiffness Parameters

IBD patients exhibited statistically significantly higher values of cAIx (16.36 ± 9.95 vs. 10.31 ± 8.19 %, *p <* 0.001), cAIx-75 (14.88 ± 10.59 vs. 6.87 ± 9.50 %, *p <* 0.001), PWV (8.06 ± 3.23 vs. 6.42 ± 1.47 m/s, *p <* 0.001) and heart rate (72.04 ± 12.21 vs. 68.13 ± 9.14 bpm, *p =* 0.027) in comparison to control group, while there was no difference in other parameters of arterial stiffness. Importantly, differences in all the aforementioned parameters were still significant after adjustment for age and BMI (*p <* 0.05). Furthermore, IBD group had significantly higher proportion of subjects with end-organ damage marked by PWV>10 m/s (17.5%, *n =* 14 vs. 2.7%, *n =* 1, *p =* 0.002) (Table 4) [40]. This pattern persisted in analysis using specific age-adjusted PWV reference values (13.8%, *n =* 11 in IBD group vs. 0.0%, *n =* 0 in control group) (Supplementary Table S7) [42]. Furthermore, participant group with positive PWV criteria for end-organ damage (PWV > 10 m/s) had significantly higher CST levels (20, 7–23 vs. 7, 4–11 ng/mL, *p =* 0.001) (Supplementary Table S5).

Finally, there was no significant difference in arterial stiffness parameters between UC and CD (Supplementary Table S8). Moreover, there was no difference in arterial stiffness parameters between IBD subgroups based on biological therapy (*p* > 0.05) (Supplementary Table S6).

### 3.5. Bivariate Correlation Analysis of CST and Selected Parameters

CST exhibited statistically significant correlation with age (*r =* 0.18, *p =* 0.023), cAIx-75 (*r =* 0.19, *p =* 0.003), PWV (*r =* 0.37, *p <* 0.001) and disease duration (*r =* -0.24, *p =* 0.032) while there was no significant correlation between CST and other variables (Table 5). There was no significant association of disease duration and PWV, but WBC positively correlated with PWV (*r =* 0.24, *p =* 0.031 and *r =* 0.12, *p =* 0.352, respectively).

### 3.6. Multiple Linear Regression Analysis of Selected Parameters as Independent Predictors of CST

The multiple linear regression model showed that PWV was the only independent predictor of CST levels (*B =* 1.20, *p <* 0.001). Other variables included in the model did not reach statistical significance (Table 6).

### 3.7. Odds for Selected Variables in Prediction of the IBD Status

Binomial logistic regression revealed that CST (1.089 (1.022–1.161), *p =* 0.009), PWV (1.515 (1.166–1.968), *p =* 0.002), cAIx-75 (1.060 (1.024–1.097), *p =* 0.001), hs-CRP (1.458 (1.116–1.906), *p =* 0.006) and BMI (0.793 (0.683–0.920), *p =* 0.002) were significant predictors of positive IBD status (Table 7).

## 4. Discussion

This study has shown that IBD patients had significantly higher levels of serum CST and arterial stiffness parameters in comparison to healthy subjects. Correspondingly, CST and PWV proved to be independent predictors of positive IBD status. In addition, an independent positive correlation exists between CST and PWV which could emphasize the relationship between IBD, CST and increased arterial stiffness. However, the role of CST in the pathophysiology of IBD and a complex link between IBD and increased cardiovascular risk have not been well-established [9,14,43].

To the best of our knowledge, only one study thus far has measured serum CST levels in a small sample of IBD patients. However, serum CST concentrations were not their primary outcome and other relevant clinical information, as well as biochemical parameters are lacking in that study [31]. Therefore, we believe that this study is the first clinical study to investigate serum CST levels in the IBD population and control subjects with detailed relevance to disease characteristics. In addition, this is the first study to analyze the relationship of CST and arterial stiffness parameters in the IBD population.

It has been previously shown that inflammatory process affects the secretion of enterochromaffine cells in the gut of IBD patients [20]. Accordingly, it seems that ChgA, a precursor of CST, is increased in colon and plasma in patients with IBD [31,44,45]. 

Derivatives of ChgA have been also connected to the pathophysiology of gastrointestinal diseases. Importantly, animal studies have shown that CST levels are significantly increased in experimental colitis model. In fact, it seems that ChgA and CST expression increases simultaneously during the iatrogenous development of collitis [31].

Findings of this study indicate that IBD patients have increased serum levels of CST in comparison to healthy subjects. Additionally, we have found that CST is an independent predictor of the IBD status. Similar to this study, Rabbi et al. found significantly higher levels of serum CST in a small sample of IBD patients [31]. However, they did not establish any difference in CST and ChgA levels between the different IBD subgroups, whereas we have found significantly higher concentrations of serum CST in UC patients [31]. Based on the existing literature, it is hard to detect reasons for this discrepancy, but it is possible that these differences in CST levels between UC and CD could be accounted for disparity in different disease activity. Specifically, UC patients had higher disease activity compared to the CD group in the present study. On contrary, study by Eissa et al. showed significantly decreased expression of CST in colonic biopsy specimens of the UC patients compared to control subjects [32]. Additionally, there was no difference in CST, PWV and cAIx-75 between the different disease activity subgroups of UC and CD. However, these results should be interpreted with caution, due to the relatively small number of cases in each subgroup. Therefore, further studies are necessary in order to confirm the role of CST in the pathophysiology of UC and CD.

Previous studies on animal models have demonstrated that CST could have a beneficial therapeutic role in IBD [29,30,31,32]. Specifically, experimental animal studies have shown that CST could have a modulatory role in inflammatory response. It has been suggested that CST could attenuate the intensity of inflammation in IBD and even possibly prevent relapse of the disease [30]. Consistently, CST delayed the onset of induced-colitis in murine models in a dose-dependent way [31,32]. Furthermore, it has been shown that the intrarectal administration of CST improves stool consistency and decreases hematochezia in murine colitis models [32]. CST treatment also significantly reduced disease severity with improved macroscopic and histologic disease scores [31,32]. These effects have been mainly mediated by local effects such as decreased colonic mucosal inflammation, edema and cellular infiltration. Similarly, we did not establish any significant correlation between CST and body weight/BMI in this study.

Several mechanisms have been proposed for these positive effects of CST on gut media in IBD. Primarily, CST induces a decrease in colonic M1 macrophages, local concentrations of proinflammatory cytokines (IL-1β, IL-6, TNF-α), activity of myeloperoxidase and CRP in animal models [30,31]. Moreover, colonic expression of CST was negatively correlated with proinflammatory cytokines interleukin 8 (IL-8) and interleukin 18 (IL-18), while CST therapy reduced IL-18 levels in UC patients [32]. Interestingly, in vivo and in vitro studies have shown that CST treatment only decreased proinflammatory cytokine and macrophage levels without any effect on anti-inflammatory cytokines and M2 macrophages [30]. Aforementioned findings could be the reason why CST treatment did not have any effect on gut histology and cytokine levels in animal models without colitis [31]. A possible mediator in this anti-inflammatory action is STAT3, a well-known factor in the pathophysiology of IBD [46]. Mouse models who were treated with high-dose CST exhibited significantly higher levels of phosphorylated STAT3 in the colon, but absolute STAT3 levels were not affected [24,25]. Importantly, when STAT3 blocker was added, cytokine secretion from the macrophages in experimental colitis was not decreased by CST [31,32]. Furthermore, it has been shown that CST therapy maintains steady levels of tight junction proteins which are a crucial indicators of cell membrane homeostasis in the gut [32,47]. Additionally, studies have shown that CST has the ability to influence the chemotaxis of monocytes [48]. Therefore, it appears that CST could yield immunomodulatory effects in the gut; however, further studies are necessary [49,50]. 

Several studies have shown that IBD patients have a higher cardiovascular risk in comparison to the general population, even though they have a lower prevalence of traditional cardiovascular risk factors [4,5,51]. This has been confirmed in this study by obtaining increased arterial stiffness and lower values of lipid parameters in IBD subjects. Arterial stiffness is a well-established predictor of the cardiovascular mortality and morbidity [42]. Findings of increased arterial stiffness in IBD patients compared to healthy subjects are consistent with most previous studies [9,52]. A pioneer study in 2012 on IBD patients showed increased arterial stiffness in comparison to healthy subjects [53]. Importantly, meta-analysis by Zanoli et al. confirmed significantly increased carotid-femoral PWV in IBD patients [9]. Consequently, IBD patients in the present study had significantly higher prevalence of end-organ damage detected by PWV > 10 m/s. 

Complex pathophysiology responsible for this paradoxical association of IBD and cardiovascular risk has not been well-determined. CST has been implicated in the pathophysiology of cardiovascular diseases and studies suggest that it has vasodilatative and antihypretensive properties [11]. Furthermore, anti-atherosclerotic, antihypertensive and cardioprotective effects have been demonstrated in previous studies [10,16,19]. Therefore, it could be possible that CST has a mediatory role in the interconnection of IBD and cardiovascular events. Results of the present study show significant negative correlation of disease duration and CST. However, subgroup comparison based on median disease duration did not show significant difference in CST. Taking into account the relatively small number of patients, future cohort longitudinal studies are necessary to elucidate the real association of disease duration and CST levels. The present findings indicate that PWV, the most important marker of arterial stiffness, is independently positively associated with serum CST levels. Importantly, PWV was the only independent predictor of CST levels. Consistently, subgroup of subjects with markers of end-organ damage have significantly higher levels of CST. Supporting findings were reported in a study on the general Japanese population, which found positive association of CST and PWV [43]. Considering that endothelial dysfunction, one of the key mechanisms for increased arterial stiffness, is largely precipitated by systemic inflammation, and that CST has speculated immunomodulatory roles, it is possible that CST could have a role in the interconnection of IBD and cardiovascular diseases [3]. From another aspect, sympathetic nervous system (SNS) activity might be an important contributor to pathologic arterial remodeling and this might translate to increased arterial stiffness parameters as postulated in previous clinical studies [54,55,56]. Importantly, sympathetic overactivity is present in patients with ulcerative colitis [57,58]. Since CST is an important antiadrenergic peptide secreted peripherally in a compensatory fashion to counterbalance catecholamine surge, elevated CST levels might simply reflect increased SNS activation and CST might be the surrogate marker of this activity. This might partially explain the high circulating CST levels in this population along with the observed increased parameters of arterial stiffness. Of note, CST and other peptides that are derived from ChgA are co-stored and co-released together with catecholamines from the storage vesicles of adrenal chromaffin cells and adrenergic neurons while circulating norepinephrine levels paralleled CST levels in a concentration-dependent manner [59]. Since the physiological effects of norepinephrine and SNS mediators promote tachyphylaxis and systemic vasoconstriction it is possible that these effects contributed to detrimental vascular remodeling as evident in increased arterial stiffness parameters. Finally, although CST stand-alone generally confers protective effects on heart and vasculature, as shown in a handful of previous studies [22,23,60,61], it is possible that in the setting of IBD, a disease state characterized by persistent inflammation and increased SNS activity, effects of CST, despite being elevated in circulation, are not sufficient to compensate for adverse arterial remodeling that is propagated by the proinflammatory and adrenergic cellular pathways. However, due to a substantial evidence gap in this regard and lack of studies reporting on CST and arterial stiffness parameters, these hypothetical interactions need to be confirmed in future preclinical and mechanistic studies.

We did not establish any difference in arterial stiffness parameters between IBD subgroups, which is consistent with the meta-analysis by Zanoli et al. and pilot research on the pediatric IBD population [9,62]. Furthermore, there was no difference in CST and arterial stiffness between different groups according to the anti-TNF-α therapy in the present study. However, there is evidence of lower PWV in IBD patients on anti-TNF-α therapy [9]. Finally, consistent with this study, the meta-analysis by Zanoli et al. did not find any difference in PWV between IBD patients with active disease and remission [9].

Interesting findings were reported by Zanoli et al. in a recently published individual participant data meta-analysis, indicating that PWV was associated with disease duration and white blood cell count [63]. On contrary, the present research did not reveal any association between disease duration and PWV but showed significant positive correlation of white blood cell and PWV which is in accordance to previously mentioned findings. These results confirm the important interaction of systemic inflammation and arterial stiffness in IBD patients.

This study has several limitations. First, the design of the present study was cross-sectional, which affects the establishment of true causal relationship. Second, this study was conducted as a single-center study and had a relatively small number of participants. The IBD group from our study mostly was heterogenous in disease activity which additionally decreases the sample size by stratification and impedes the generalization of the findings to the entire IBD population. Moreover, as IBD is relapsing and remitting disease, secretion pattern of biomarkers is not constant and therefore one single measurement cannot be an ideal strategy for determination of causal relationship. Furthermore, any time dependent effects of IBD cannot be truly observed by our study model. Additionally, we did not measure all inflammatory parameters which could have an important role in clarifying some of the results, except hs-CRP. Finally, direct markers of sympathetic nervous system activity, such as circulating catecholamines or urinary catecholamines, were not measured in this study due to financial constraints and thus they are only indirectly discussed. 

## 5. Conclusions

In conclusion, serum CST levels and arterial stiffness parameters are significantly increased in IBD patients compared to healthy subjects, which could indicate a higher cardiovascular risk in this population. However, future observational studies are necessary to explain complex pathophysiology of CST and arterial stiffness in the IBD population. 

## Figures and Tables

**Table 1:** Basic characteristics of IBD and control group.

Parameter		IBD Group (*n* = 80)	Control Group (*n* = 75)	*p*
Age (years)		39.79 ± 14.42	38.47 ± 12.22	0.543 *
Male gender		50 (62.5%)	46 (61.3%)	0.987 **
Body weight (kg)		75.03 ± 14.10	80.47 ± 13.28	0.015 *
Body height (cm)		176.41 ± 9.27	180.03 ± 9.18	0.017 *
BMI (kg/m^2^)		24.04 ± 3.83	24.69 ± 2.75	0.231 *
BMI categories	Underweight (<18.5 kg/m^2^)	3 (3.7%)	0 (0.0%)	0.187 ***
	Normal weight (18.5–24.9 kg/m^2^)	39 (48.8%)	42 (56.0%)	
	Overweight (25.0–29.9 kg/m^2^)	36 (45.0%)	30 (40.0%)	
	Obesity class I (30.0–34.9 kg/m^2^)	2 (2.5%)	3 (4.0%)	
Waist-hip ratio		0.9 (0.8–0.9)	0.9 (0.8–0.9)	0.172 ****
Positive family history on CRC		13 (16.3%)	10 (13.3%)	0.776 **
Positive family history on IBD		16 (20.0%)	3 (4.0%)	0.003 ***
Positive family history on CV disease		29 (36.2%)	36 (48.0%)	0.187 **
Alcohol consumption		23 (28.7%)	31 (41.3%)	0.090 **
Coffee consumption		41 (51.2%)	44 (58.7%)	0.354 **
Active smoking		19 (23.7%)	12 (16.4%)	0.356 **

Continuous parametric data are presented as mean ± standard deviation for parametric data, continuous non-parametric data are presented as median (interquartile range) and categorical data are presented as number (percentage). IBD: inflammatory bowel disease; BMI: body mass index; CRC: colorectal cancer; CV: cardiovascular. * Student t-test for independent samples. ** Chi-square test. *** Fisher’s exact test. **** Mann–Whitney U test.

**Table 2:** Basic anthropometric and disease characteristics of UC and CD group.

Parameter		Ulcerative Colitis (*n* = 35)	Crohn’s Disease (*n* = 45)	*p*
Age (years)		42.66 ± 15.57	37.56 ± 13.38	0.119 *
Male gender		21 (60.0%)	29 (64.4%)	0.684 **
Body weight (kg)		77.31 ± 13.03	73.26 ± 14.92	0.206 *
Body height (cm)		176.29 ± 8.47	176.51 ± 10.03	0.915 *
BMI (kg/m^2^)		24.90 ± 3.98	23.38 ± 3.65	0.079 *
BMI categories	Underweight (<18.5 kg/m^2^)	2 (5.7%)	1 (2.2%)	0.093 ***
	Normal weight (18.5–24.9 kg/m^2^)	14 (40.0%)	25 (55.6%)	
	Overweight (25.0–29.9 kg/m^2^)	18 (51.4%)	18 (40.0%)	
	Obesity class I (30.0–34.9 kg/m^2^)	1 (2.9%)	1 (2.2%)	
Waist-hip ratio		0.9 (0.8–1.0)	0.9 (0.8–0.9)	0.284 ****
SES-CD		-	10 (5–15)	-
CDAI		-	57 (32–102)	-
HBI		-	2 (1–4)	-
UCEIS		6 (5–7)	-	-
MES		2 (2–3)	-	-
Mayo/DAI		4 (2–7)	-	-
Disease duration (years)		9 (5–11)	6 (3–13)	0.268 ****
Positive family history on CRC		6 (17.1%)	7 (15.6%)	0.849 **
Positive family history on IBD		7 (20.0%)	9 (20.0%)	1.000 **
Positive family history on CV disease		11 (31.4%)	18 (40.0%)	0.429 **
Alcohol consumption		9 (25.7%)	14 (31.1%)	0.597 **
Active smoking		7 (20.0%)	12 (26.7%)	0.487 **
IBD-related surgical procedures		1 (2.9%)	16 (35.6%)	<0.001 ***
Extraintestinal manifestations		6 (18.2%)	27 (62.8%)	<0.001 **
Aminosalicylates		30 (85.7%)	27 (60.0%)	0.012 **
DMARD		12 (34.3%)	11 (24.4%)	0.335 **
Monoclonal antibodies		21 (60.0%)	30 (66.7%)	0.538 **

Continuous parametric data are presented as mean ± standard deviation, continuous non-parametric data are presented as median (interquartile range) and categorical data are presented as number (percentage). BMI: body mass index; SES-CD: simple endoscopic score for Crohn’s disease; CDAI: Crohn’s disease activity index; HBI: Harvey-Bradshaw index; UCEIS: ulcerative colitis endoscopic index of severity; MES: Mayo endoscopic score; Mayo/DAI: Mayo score/disease activity index for ulcerative colitis; CRC: colorectal cancer; IBD: inflammatory bowel disease; CV: cardiovascular; DMARD: disease modifying antirheumatic drugs. * Student t-test for independent samples. ** Chi-square test. *** Fisher’s exact test. **** Mann–Whitney U test.

**Table 3:** Comparison of serum CST concentrations between the study groups.

Parameter	Study Groups		Test Type	
CST (ng/mL)	**IBD (*n =* 80)**	**Control group (*n =* 75)**	p *	p **
	11.29 ± 9.14	7.13 ± 6.08	0.001	0.001
	**Ulcerative colitis (*n =* 35)**	**Crohn’s disease (*n =* 45)**	p *	p **
	13.50 ± 9.58	9.03 ± 6.92	0.021	0.036

Data are presented as mean ± standard deviation. CST: catestatin. * Student t-test for independent samples. ** ANCOVA model adjusted for age and BMI.

**Table 4:** Comparison of selected arterial stiffness parameters between IBD and control group.

Parameter	IBD Group (*n =* 80)	Control Group (*n =* 75)	*p* *	p **
pSBP (mmHg)	120.63 ± 7.15	118.57 ± 7.32	0.080	0.058
pDBP (mmHg)	74.44 ± 6.71	73.39 ± 9.16	0.419	0.483
pMBP (mmHg)	89.78 ± 5.98	88.44 ± 6.96	0.201	0.194
pPP (mmHg)	46.19 ± 7.17	45.19 ± 10.67	0.497	0.333
cSBP (mmHg)	106.13 ± 6.97	104.37 ± 6.43	0.106	0.087
cDBP (mmHg)	75.29 ± 6.54	74.16 ± 6.49	0.283	0.264
cMBP (mmHg)	85.55 ± 5.99	84.24 ± 6.17	0.182	0.154
cPP (mmHg)	30.84 ± 6.27	30.21 ± 3.98	0.458	0.420
HR (bpm)	72.04 ± 12.21	68.13 ± 9.14	0.027	0.037
pAIx (%)	−37.30 ± 16.13	−40.41 ± 15.48	0.223	0.529
cAIx (%)	16.36 ± 9.95	10.31 ± 8.19	<0.001	0.005
cAIx-75 (%)	14.88 ± 10.59	6.87 ± 9.50	<0.001	0.003
PWV (m/s)	8.06 ± 3.23	6.42 ± 1.47	<0.001	<0.001
End-organ damage ^a^	14 (17.5%)	2 (2.7%)	0.002 ***	n/a

Continuous data are presented as mean ± standard deviation and categorical data are presented as number (percentage). pSBP: peripheral systolic blood pressure; pDBP: peripheral diastolic blood pressure; pMBP: peripheral mean blood pressure; pPP: peripheral pulse pressure; cSBP: central systolic blood pressure; cDBP: central diastolic blood pressure; cMBP: central mean blood pressure; cPP: central pulse pressure; HR: heart rate; bpm: beats per minute; pAIx: peripheral augmentation index; cAIx: central augmentation index; cAIx-75: central augmentation index corrected for heart rate; PWV: pulse wave velocity. * Student t-test for independent samples. ** ANCOVA model adjusted for age and BMI. *** Chi-square test. ^a^ According to the ESC/EHA guidelines, PWV > 10 m/s is considered to represent an end-organ damage [40].

**Table 5:** Correlation between parameters of CTS and selected parameters.

r/ρ * (*p*)																					
**Anthropometric parameters**																					
**Age**				**BMI**					**Body weight**				**Body height**					**Waist-hip ratio**			
0.18 (0.023 **)				0.15 (0.059 **)					−0.01 (0.919 **)				−0.16 (0.050 **)					0.16 (0.058 **)			
**hs-CRP, WBC and lipid parameters**																					
**hs-CRP**		**Cholesterol**				**TG**				**LDL**					**HDL**				**WBC**		
−0.07 (0.393 **)		0.10 (0.193 **)				0.01 (0.894 **)				0.06 (0.420 **)					0.12 (0.142 **)				0.03 (0.715 **)		
**Arterial stiffness parameters**																					
**HR**	**cSBP**				**cDBP**			**cMBP**			**cPP**			**pAIx**			**cAIx-75**				**PWV**
0.11 (0.172 **)	0.11 (0.161 **)				0.08 (0.346 **)			0.09 (0.251 **)			0.13 (0.105 **)			0.10 (0.209 **)			0.19 (0.003 **)				0.37 (<0.001 **)
**IBD-related parameters ** ****																					
**CD**			**UC**													**Disease duration**				**FC**	
**SES-CD**			**UCEIS**				**MES**					**Mayo/DAI**									
0.20 (0.188 ***)			0.19 (0.285 ***)				0.04 (0.812 ***)					0.05 (0.776 ***)				−0.24 (0.032 ***)				0.07 (0.531 **)	

BMI: body mass index; hs-CRP: high sensitivity C-reactive protein; TG: triglycerides; LDL: low density lipoproteins; HDL: high density lipoproteins; WBC: white blood cell; HR: heart rate; cSBP: central systolic blood pressure; cDBP: central diastolic blood pressure; cMBP: central mean blood pressure; cPP: central pulse pressure; pAIx: peripheral augmentation index; cAIx-75: central augmentation index corrected for heart rate; PWV: pulse wave velocity; SES-CD: simple endoscopic score for Crohn’s disease; UCEIS: ulcerative colitis endoscopic index of severity; MES: Mayo endoscopic score; Mayo/DAI: Mayo score/disease activity index for ulcerative colitis; FC: fecal calprotectin. * r/ρ-value indicates correlation coefficient. ** Pearson’s correlation test. *** Spearman’s correlation test. **** Correlation in the IBD group.

**Table 6:** Multiple linear regression analysis of selected variables as independent predictors of CST.

Parameter	*B* * (*t* **) *p*	Overall
**Age**	−0.07 (−1.11) 0.270	*R*^2^ adjusted = 0.133 F ratio = 5.739 *p <* 0.001
**BMI**	0.10 (0.50) 0.620	
**Waist-hip ratio**	0.36 (1.32) 0.189	
**cAIx-75**	0.07 (1.68) 0.095	
**PWV**	1.20 (4.15) <0.001	

BMI: body mass index; cAIx-75: central augmentation index corrected for heart rate; PWV: pulse wave velocity. * B-value indicates regression coefficient of the independent variable. ** t-value indicates t-statistic value.

**Table 7:** Binomial logistic regression for the prediction of IBD status.

Predictor	OR (95% CI)	*p*
**CST**	1.089 (1.022–1.161)	0.009
**BMI**	0.793 (0.683–0.920)	0.002
**PWV**	1.515 (1.166–1.968)	0.002
**cAIx-75**	1.060 (1.024–1.097)	0.001
**hs-CRP**	1.458 (1.116–1.906)	0.006

CST: catestatin; BMI: body mass index; PWV: pulse wave velocity; cAIx-75: central augmentation index corrected for heart rate; hs-CRP: high sensitivity C-reactive protein.

## Supplementary Materials

The following are available online at https://www.mdpi.com/2077-0383/9/3/628/s1, Figure S1: Flow diagram, Table S1: Detailed laboratory characteristics of IBD and control group, Table S2: Detailed laboratory characteristics of UC and CD group, Table S3: Comparison of CST levels between IBD subgroups according to median of disease duration and PWV thresholds for end-organ damage, Table S4: Comparison of CST levels between IBD subgroups according to biologic therapy, Table S5: Prevalence of abnormal age-adjusted PWV valuess among studied groups, Table S6: Comparison of selected arterial stiffness parameters between UC and CD subgroup.
