# Supplementary material for: Serum Catestatin Levels and Arterial Stiffness Parameters Are Increased in Patients with Inflammatory Bowel Disease
Source: J Clin Med. 2020 Feb 26;9(3):628. doi: 10.3390/jcm9030628 (PMC7141110; doi:10.3390/jcm9030628)
Supplement: Supplementary file 1 [file jcm-09-00628-s001.pdf]

Table S1. Detailed laboratory characteristics of IBD and control group

Parameter	IBD group (n=80)	Control group (n=75)	*p**
**RBC (x10^{12}/L)**	4.77 ± 0.56	5.07 ± 0.43	<0.001
**Hb (g/L)**	138.09 ± 18.90	147.95 ± 14.63	<0.001
**Hct (L/L)**	0.42 ± 0.05	0.45 ± 0.04	<0.001
**WBC (x10^{9}/L)**	8.99 ± 11.68	6.46 ± 1.45	0.064
**Platelets (x10^{9}/L)**	260.99 ± 74.56	240.53 ± 58.52	0.060
**hs-CRP (mg/L)**	10.26 ± 9.48	1.25 ± 1.21	<0.001
**Fating glucose (mmol/L)**	5.28 ± 1.85	5.11 ± 0.65	0.457
**Urea (mmol/L)**	4.57 ± 1.40	5.51 ± 1.58	<0.001
**Creatinine (μmol/L)**	71.49 ± 14.49	76.67 ± 14.71	0.030
**Uric acid (μmol/L)**	276.31 ± 72.37	296.59 ± 75.01	0.091
**Bilirubin total (μmol/L)**	12.59 ± 7.38	14.85 ± 7.95	0.069
**Bilirubin conjugated (μmol/L)**	4.75 ± 3.36	4.89 ± 2.03	0.752
**AST (U/L)**	25.49 ± 40.91	20.96 ± 8.91	0.353
**ALT (U/L)**	27.08 ± 37.48	26.43 ± 16.03	0.891
**GGT (U/L)**	25.79 ± 25.77	21.17 ± 11.58	0.160
**LDH (U/L)**	170.39 ± 52.01	161.89 ± 25.18	0.205
**ALP (U/L)**	72.64 ± 37.09	61.32 ± 16.74	0.017
**Iron (μmol/L)**	15.46 ± 8.11	18.11 ± 6.61	0.029
**Ferritin (μg/L)**	69.10 ± 101.37	85.43 ± 72.95	0.257
**Proteins (g/L)**	71.89 ± 7.40	72.71 ± 3.71	0.393
**Albumins (g/L)**	39.28 ± 5.01	43.92 ± 2.52	<0.001
**Cholesterol total (mmol/L)**	4.80 ± 1.43	5.27 ±1.16	0.028
**Triglycerides (mmol/L)**	1.38 ± 1.16	1.21 ± 0.63	0.266
**LDL (mmol/L)**	2.81 ± 1.13	3.29 ± 1.04	0.006
**HDL (mmol/L)**	1.33 ± 0.43	1.42 ± 0.31	0.159
**FC (mg/kg)**	513.94 ± 864.87	n/a	n/a

Data are presented as mean ± standard deviation.

RBC – red blood cells; Hb – hemoglobin; Hct – hematocrit; WBC – white blood cells; hs-CRP – high sensitivity C-reactive protein; AST – aspartate aminotransferase; ALT – alanine aminotransferase; GGT – gamma-glutamyl transferase; LDH – lactate dehydrogenase; ALP – alkaline phosphatase; LDL – low density lipoproteins; HDL – high density lipoproteins; FC – fecal calprotectin.

*Student t-test for independent samples.

Table S2. Comparison of selected parameters between different disease activity categories in UC patients

Parameters	Endoscopic disease activity (UCEIS; MES)		p*
	**Moderate (N=18)**	**Severe (N=15)**	
CST (ng/mL)	13 (6-18)	14 (5-20)	0.850
PWV (m/s)	7 (6-11)	7 (6-10)	0.694
cAIx-75 (%)	12 (8-26)	16 (10-25)	0.906

Data were presented as median (interquartile range).
UCEIS – ulcerative colitis endoscopic index of severity; MES – Mayo endoscopic score; CST – catestatin; PWV – pulse wave velocity; cAIx-75 – central augmentation index corrected for heart rate.
*Mann Whitney U test

Table S3. Comparison of selected parameters between different disease activity categories in CD patients

Parameters	Endoscopic disease activity (SES-CD)			p*
	**Remission (N=7)**	**Mild (N=10)**	**Moderate (N=27)**	
CST (ng/mL)	10 (5-17)	11 (8-18)	11 (6-17)	0.861
PWV (m/s)	8 (6-12)	6 (4-11)	7 (5-13)	0.901
cAIx-75 (%)	15 (7-19)	15 (9-19)	17 (8-21)	0.253

Data were presented as median (interquartile range).
SES-CD – simple endoscopic score for Crohn’s disease; CST – catestatin; PWV – pulse wave velocity; cAIx-75 – central augmentation index corrected for heart rate.
*Mann Whitney U test

Table S4. Detailed laboratory characteristics of UC and CD group

Parameter	Ulcerative colitis (n=35)	Crohn's disease (n=45)	*p**
**RBC (x10[12]/L)**	4.81 ± 0.65	4.74 ± 0.48	0.555
**Hemoglobin (g/L)**	140.09 ± 20.15	136.53 ± 17.95	0.408
**Hematocrit (L/L)**	0.42 ± 0.06	0.42 ± 0.05	0.449
**WBC (x10[9]/L)**	10.45 ± 17.33	7.86 ± 3.10	0.328
**Platelets (x10[9]/L)**	241.09 ± 59.66	276.47 ± 81.68	0.034
**hs-CRP (mg/L)**	2.33 ± 2.19	16.43 ± 12.93	<0.001
**Fating glucose (mmol/L)**	5.65 ± 2.67	4.99 ± 0.73	0.114
**Urea (mmol/L)**	4.55 ± 1.39	4.58 ± 1.44	0.947
**Creatinine (µmol/L)**	70.97 ± 10.77	71.90 ± 17.09	0.780
**Uric acid (µmol/L)**	280.91 ± 58.27	272.73 ± 82.88	0.621
**Bilirubin total (µmol/L)**	13.06 ± 5.58	12.22 ± 8.64	0.621
**Bilirubin conjugated (µmol/L)**	4.83 ± 2.72	4.69 ± 3.84	0.856
**AST (U/L)**	23.49 ± 25.48	27.04 ± 50.35	0.704
**ALT (U/L)**	26.34 ± 32.26	27.64 ± 41.82	0.879
**GGT (U/L)**	27.60 ± 25.06	24.38 ± 26.79	0.585
**LDH (U/L)**	167.23 ± 39.60	172.84 ± 60.77	0.637
**ALP (U/L)**	71.54 ± 34.13	73.49 ± 39.99	0.819
**Iron (µmol/L)**	16.98 ± 8.01	14.27 ± 8.16	0.141
**Ferritin (µg/L)**	50.77 ± 48.22	83.36 ± 128.11	0.158
**Proteins (g/L)**	73.51 ± 5.54	70.62 ± 8.49	0.085
**Albumins (g/L)**	40.96 ± 3.91	37.96 ± 5.46	0.008
**Cholesterol total (mmol/L)**	5.42 ± 1.41	4.31 ± 1.27	<0.001
**Triglycerides (mmol/L)**	1.15 ± 0.70	1.56 ± 1.42	0.122
**LDL (mmol/L)**	3.37 ± 1.19	2.37 ± 0.88	<0.001
**HDL (mmol/L)**	1.45 ± 0.43	1.24 ± 0.42	0.031
**FC (mg/kg)**	366.29 ± 515.09	628.78 ± 1060.27	0.183

Data are presented as mean ± standard deviation.

RBC – red blood cells; Hb – hemoglobin; Hct – hematocrit; WBC – white blood cells; hs-CRP – high sensitivity C-reactive protein; AST – aspartate aminotransferase; ALT – alanine aminotransferase; GGT – gamma-glutamyl transferase; LDH – lactate dehydrogenase; ALP – alkaline phosphatase; LDL – low density lipoproteins; HDL – high density lipoproteins; FC – fecal calprotectin.

*Student t-test for independent samples.

Table S5. Comparison of CST levels between IBD subgroups according to median of disease duration and PWV thresholds for end-organ damage

**Parameter**			***p****
**CST (ng/mL)**	**Disease duration**		
	**≤ 9 years (n=51)**	**> 9 years (n=29)**	
	10 (5-18)	7 (4-7)	0.180
	**PWV**		
	**≤ 10 m/s (n=139)**	**> 10 m/s (n=16)**	
	7 (4-11)	20 (7-23)	0.001

Data are presented as median (interquartile range).

CST – catestatin, PWV – pulse wave velocity.

*Mann Whitney U test.

Table S6. Comparison of CST levels between IBD subgroups according to biologic therapy

Parameter	Biological therapy (N=51)	Non-biological therapy (N=29)	*p**
**CST (ng/mL)**	11.21 ± 8.22	11.42 ± 10.72	0.923
**pSBP (mmHg)**	120.73 ± 7.07	120.45 ± 7.42	0.869
**pDBP (mmHg)**	73.76 ± 6.76	75.62 ± 6.56	0.236
**pMBP (mmHg)**	89.42 ± 6.07	90.56 ± 5.82	0.413
**pPP (mmHg)**	46.96 ± 6.80	44.83 ± 7.70	0.203
**cSBP (mmHg)**	106.16 ± 6.63	106.07 ± 7.63	0.957
**cDBP (mmHg)**	75.16 ± 6.68	75.52 ± 6.40	0.814
**cMBP (mmHg)**	85.49 ± 5.72	85.70 ± 6.55	0.881
**cPP (mmHg)**	31.00 ± 7.26	30.55 ± 4.11	0.761
**HR (bpm)**	72.24 ± 11.81	71.69 ± 13.07	0.849
**pAIx (%)**	-35.90 ± 17.66	-39.76 ± 18.29	0.356
**cAIx (%)**	17.76 ± 10.29	13.90 ± 10.19	0.110
**cAIx-75 (%)**	16.38 ± 9.24	13.24 ± 11.03	0.177
**PWV (m/s)**	8.25 ± 3.27	7.72 ± 3.18	0.488

Data are presented as mean ± standard deviation.

CST – catestatin; pSBP – peripheral systolic blood pressure; pDBP – peripheral diastolic blood pressure; pMBP – peripheral mean blood pressure; pPP – peripheral pulse pressure; cSBP – central systolic blood pressure; cDBP – central diastolic blood pressure; cMBP – central mean blood pressure; cPP – central pulse pressure; HR – heart rate; bpm – beats per minute; pAIx – peripheral augmentation index; cAIx – central augmentation index; cAIx-75 – central augmentation index corrected for heart rate; PWV – pulse wave velocity.

*Student t-test for independent samples.

Table S7. Prevalence of abnormal age-adjusted PWV valuess among studied groups

Age group (PWV)[a]	IBD group (n=80)	Control group (n=75)	*p**
**<30 years (>8.2 m/s)**	3 (18.8%)	0 (0.0%)	0.064
**30-39 years (>9.4 m/s)**	1 (6.3%)	0 (0.0%)	0.295
**40-49 years (>10.0 m/s)**	3 (15.0%)	0 (0.0%)	0.143
**50-59 years (>11.7 m/s)**	1 (10.0%)	0 (0.0%)	0.262
**60-69 years (>13.6 m/s)**	3 (50.0%)	0 (0.0%)	0.064

Data are presented as number and percentages.

IBD – inflammatory bowel disease; PWV – pulse wave velocity.

*Fisher test.

[a]Age-adjusted upper reference limit for PWV in subjects with normal blood pressure values [39].

Table S8. Comparison of selected arterial stiffness parameters between UC and CD subgroup

Parameter	Ulcerative colitis (n=35)	Crohn's disease (n=45)	*p**	*p***
**pSBP (mmHg)**	120.46 ± 7.58	120.76 ± 6.89	0.854	0.892
**pDBP (mmHg)**	75.66 ± 6.60	73.49 ± 6.71	0.153	0.278
**pMBP (mmHg)**	90.49 ± 6.25	89.22 ± 5.76	0.352	0.498
**pPP (mmHg)**	44.80 ± 6.32	47.27 ± 7.66	0.128	0.236
**cSBP (mmHg)**	106.14 ± 7.92	106.11 ± 6.22	0.984	0.840
**cDBP (mmHg)**	75.74 ± 6.52	74.93 ± 6.61	0.586	0.777
**cMBP (mmHg)**	85.86 ± 6.34	85.31 ± 5.77	0.689	0.908
**cPP (mmHg)**	30.40 ± 6.43	31.18 ± 6.20	0.585	0.605
**HR (bpm)**	74.51 ± 12.24	70.11 ± 11.96	0.110	0.093
**pAIx (%)**	-38.00 ± 19.88	-36.76 ± 18.76	0.776	0.058
**cAIx (%)**	15.97 ± 10.81	16.67 ± 11.90	0.787	0.183
cAIx-75 (%)	16.73 ± 12.90	13.44 ± 12.47	0.628	0.439
**PWV (m/s)**	8.83 ± 4.25	7.46 ± 1.98	0.085	0.306
**End-organ damage**[a]	9 (25.7%)	5 (11.1%)	0.088***	n/a

Continuous data are presented as mean ± standard deviation and categorical data are presented as number (percentage).

pSBP – peripheral systolic blood pressure; pDBP – peripheral diastolic blood pressure; pMBP – peripheral mean blood pressure; pPP – peripheral pulse pressure; cSBP – central systolic blood pressure; cDBP – central diastolic blood pressure; cMBP – central mean blood pressure; cPP – central pulse pressure; HR – heart rate; bpm – beats per minute; pAIx – peripheral augmentation index; cAIx – central augmentation index; cAIx-75 – central augmentation index corrected for heart rate; PWV – pulse wave velocity.

*Student t-test for independent samples.

**ANCOVA model adjusted for age and BMI.

***Chi-square test.

[a]According to the ESC/EHA guidelines, PWV >10 m/s is considered to represent an end-organ damage [41].
